# Temperature Impacts the Development and Survival of Common Cutworm (*Spodoptera litura*): Simulation and Visualization of Potential Population Growth in India under Warmer Temperatures through Life Cycle Modelling and Spatial Mapping

**DOI:** 10.1371/journal.pone.0124682

**Published:** 2015-04-30

**Authors:** Babasaheb B. Fand, Nitin T. Sul, Santanu K. Bal, P. S. Minhas

**Affiliations:** ICAR-National Institute of Abiotic Stress Management (NIASM), Malegaon, Baramati, Pune, Maharashtra, India; Universidad Nacional Autonoma de Mexico, MEXICO

## Abstract

The common cutworm, *Spodoptera litura*, has become a major pest of soybean (*Glycine max*) throughout its Indian range. With a changing climate, there is the potential for this insect to become an increasingly severe pest in certain regions due to increased habitat suitability. To examine this possibility, we developed temperature-based phenology model for *S*. *litura*, by constructing thermal reaction norms for cohorts of single life stages, at both constant and fluctuating temperatures within the ecologically relevant range (15–38°C) for its development. Life table parameters were estimated stochastically using cohort updating and rate summation approach. The model was implemented in the geographic information system to examine the potential future pest status of *S*. *litura* using temperature change projections from SRES A_1_B climate change scenario for the year 2050. The changes were visualized by means of three spatial indices demonstrating the risks for establishment, number of generations per year and pest abundance according to the temperature conditions. The results revealed that the development rate as a function of temperature increased linearly for all the immature stages of *S*. *litura* until approximately 34–36°C, after which it became non-linear. The extreme temperature of 38°C was found lethal to larval and pupal stages of *S*. *litura* wherein no development to the next stage occurred. Females could lay no eggs at the extreme low (15°C) and high (> 35°C) test temperatures, demonstrating the importance of optimum temperature in determining the suitability of climate for the mating and reproduction in *S*. *litura*. The risk mapping predicts that due to temperature increase under future climate change, much of the soybean areas in Indian states like Madhya Pradesh, Maharashtra and Rajasthan, will become suitable for *S*. *litura* establishment and increased pest activity, indicating the expansion of the suitable and favourable areas over time. This has serious implication in terms of soybean production since these areas produce approximately 95% of the total soybeans in India. As the present model results are based on temperature only, and the effects of other abiotic and biotic factors determining the pest population dynamics were excluded, it presents only the potential population growth parameters for *S*. *litura*. However, if combined with the field observations, the model results could certainly contribute to gaining insight into the field dynamics of *S*. *litura*.

## Introduction

The common cutworm *Spodoptera litura* (Fabricius) (Lepidoptera: Noctuidae), is a destructive insect pest, damaging economically important crops like tobacco (*Nicotiana tabacum* L.), castor (*Ricinus communis* L.), cotton (*Gossypium* sp. L.), soybean (*Glycine max* L.) and groundnut (*Arachis hypogea* L.) throughout tropical and temperate Asia, Australasia and the Pacific Islands [1, 2]. Out of 112 globally recorded host plants of *S*. *litura* [3, 4], 60 are known only from India [5]. Due to nocturnal habit, high mobility of adult moths and ability to oviposit on a wide range of host plants, *S*. *litura* has huge potential to invade new areas and to adapt to wide range of ecological situations [6]. In India, *S*. *litura* is widespread in almost all the states and inflict significant losses to crops of economic importance like soybean [7–9], cotton [10] and groundnut [11, 12]. A single larva per square metre is reported to cause average pod yield loss of 27.3% in groundnut through damage to various plant parts like leaves, flowers and pods [11]. Since 2002, it has frequently been reported that the larvae of *S*. *litura* are causing widespread damage to soybean crops at several localities in India [7–9, 13–16]. On soybeans, the pest remains active from end of July or mid of August to October coinciding with warm and humid climate and peak reproductive phases of soybean, causing 26–29% yield losses [8]. Recent outbreaks of *S*. *litura* on soybean in Kota (Rajasthan state), and Marathwada and Vidarbha (Maharashtra state) regions of India have been reported to cause monetary losses to the tune of USD 4.5 crores and USD 22.5 crores, respectively [15, 17].

Being poikilothermic organisms, the developmental rate in insects is highly contingent on external temperature conditions. Hence, temperature is generally considered the single most significant environmental factor influencing behaviour, distribution, development, survival and reproduction in insects [18]. Knowledge on the temperature-dependent population growth potential of insect pests is highly imperative for understanding their population dynamics and implementing agro-ecoregion specific pest control strategies, especially in the context of predicted global climate warming [19–22]. Considering this, the temperature rise of 2.7–4.7°C predicted due to potential climate change [23] may have drastic consequences for future incidence of *S*. *litura*. The vast majority of studies that infer the effects of temperature on developmental biology of *S*. *litura* have been undertaken under only one constant temperature in laboratory [10, 24–31]. A few number of studies that addressed the development of *S*. *litura* at a range of constant temperatures, were concerned with predicting only developmental rates and threshold temperatures using linear degree day or heat summation models [24, 28], but no emphasis was given to the simulation of variability in development times, mortality and fecundity with temperature changes. Due to non-linearity in developmental response at temperature extremes, linear models are generally considered poor predictors of insect developmental rates [19, 32, 33]. Yet, the specific effects of associated daily and seasonal temperature extremes on *S*. *litura* development are less understood which warrants estimation of the temperature-dependent population growth potential for understanding the impact of climate change on its future incidence and damage activity.

The objective of this study was to develop a comprehensive temperature-based population model for *S*. *litura*, which permits prediction of its population growth potential and seasonal dynamics in various soybean growing regions of India, and will also aid in forecasting the probable pest aggravation under potential climate warming. The Insect Life Cycle Modelling (ILCYM) software developed by the International Potato Centre (CIP) [34] was used for the development of process-based temperature-driven and age-stage structured *S*. *litura* phenology model. ILCYM has been used to predict climatically suitable areas for distribution, abundance and damage activity, and to examine the impact of climate change on future pest status of insects of economically important crops [*e*.*g*.: potato tuber moth [22, 35], cotton mealybug [36, 37], maize stem borer [38]].The implications of the present work are multifaceted and include an assumption that any small increases in average global temperatures may have measurable effects on *S*. *litura* performance and thus on the yield losses in soybean. We contribute to the exploration of the differing developmental responses of *S*. *litura* to variability in environmental temperatures. Here, we first (1) establish thermal reaction norms to various constant temperature conditions for generating models of developmental response in *S*. *litura*, and use them to test if the developmental effects of temperature variations predicted from the models established at constant temperatures are in concordance with those observed in the experiments conducted using daily temperature fluctuations, and then (2) visualize the potential changes in pest status of *S*. *litura* due to future temperature increase by implementing the developed phenology model in the geographic information system (GIS) using climate change projections from general circulation models (GCMs) and climate change scenarios.

## Materials and Methods

### Origin of *S*. *litura* colony

During the *kharif* (rainy) seasons of 2013 and 2014, the first and second instar larvae of *S*. *litura* were collected from a soybean field of ICAR-National Institute of Abiotic Stress Management (NIASM) campus (Malegaon Khurd), Baramati near the city of Pune in the state of Maharashtra in India (Location: 18.13 N, 74.52 E, Alt. 566 m). The identity of the insect was confirmed by Dr. V.V. Ramamurthy, a taxonomist from the Insect Identification Service of the Division of Entomology, Indian Agricultural Research Institute, New Delhi (Voucher specimens RRS No. 13-16/14). The laboratory colony of *S*. *litura* was maintained on soybean leaves (cultivar JS-335) at 25 ± 1°C temperature, 70 ± 10% RH and natural photoperiod [28, 39].

### Design of Thermal Environments

#### General rearing conditions

**Fig 1:**
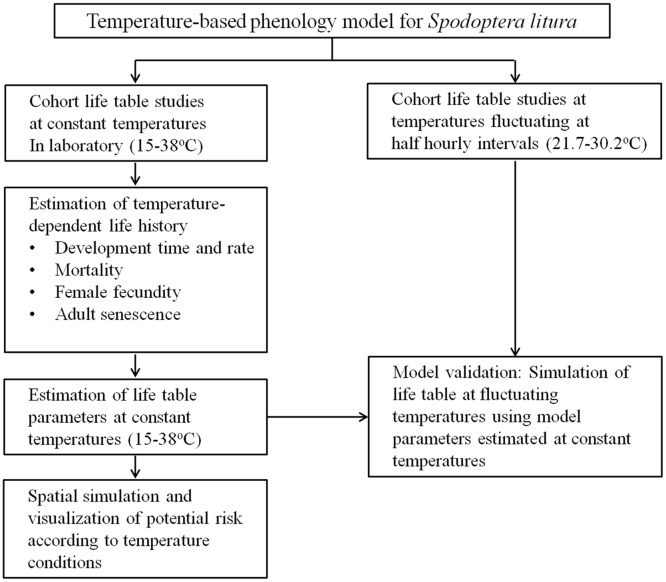
Scheme of model implementation for estimating temperature-dependent *S*. *litura* population growth.

We raised *S*. *litura* populations at six constant and one fluctuating temperature environments created in programmable incubators (Model E-36L2, M/s. Percival Scientific, USA) (Fig 1). The designed temperature treatments ranged across the natural temperature range experienced by *S*. *litura* under field conditions. The required temperatures inside the chambers were regularly monitored and the experiments in which temperatures fluctuated by >1.0°C were omitted from the analysis. The relative humidity was maintained between 60% and 80%, and photoperiod regime was kept at 10L: 14D h.

#### Constant temperature treatments

The life-tables were constructed for *S*. *litura* under six constant temperature environments between 15°C and 38°C programmed inside the controlled incubation chambers. The first five temperature treatments were designed at 5°C increments (15, 20, 25, 30 and 35°C), whereas the last temperature treatment was set up with 3°C increment (*i*.*e*. 38°C). This was chosen considering the upper threshold somewhere between 36–39°C, as *S*. *litura* development is reported to follow nonlinearity above 35°C [28]. The data obtained from these experiments were then used to develop temperature-dependent population model for *S*. *litura*.

#### Fluctuating temperature treatments

The life-table was constructed for *S*. *litura* under fluctuating temperature conditions programmed inside the controlled incubation chamber. The daily temperature fluctuations in an incubator were programmed at half hourly intervals to ramp between 21.7°C to 30.2°C every 24 hours. The DTF range was selected by averaging the half hourly temperature records for the period of June-October for two years *i*.*e*. 2012 and 2013. It represented the average half hourly temperatures during soybean growing season in Maharashtra state in India, covering the low, moderate and high temperature ranges for *S*. *litura* development. The data obtained from this experiment were used to validate the temperature-dependent population model developed for *S*. *litura* at constant temperatures.

### Data collection for cohort life tables

The effects of temperatures on the biology of *S*. *litura* were studied in cohorts of single life stages [34, 40]. Each life stage of *S*. *litura* was maintained and evaluated at six constant and one fluctuating temperatures as follows.

A week old seedlings of soybean raised in plastic cups were kept inside the moth mating jars in the evening at 6.00 PM and removed in the next day morning at 8.00 AM to ensure that all eggs of *S*. *litura* were laid during less than 14 h interval. Only one egg mass (having about 100–150 eggs) was retained per plant and excess egg masses if any, were removed by trimming the leaves holding them. Four such jars each holding a plant with single egg mass were kept inside the growth chambers for incubation at respective temperatures so as to observe at least 400–500 individual eggs at each test temperature. The number of eggs hatched from each egg mass was recorded daily. After one day of complete hatching, the tuft of anal hairs of female covering the egg mass was removed and unhatched eggs were counted. The sample size *i*.*e*. the total number of eggs in each egg mass kept for hatching was determined by adding the numbers hatched and unhatched.

A group of 25 newly hatched (< 12 h old) *S*. *litura* larvae from stock colony was transferred to plastic jar (30 x 15 cm size) and was kept for incubation in the growth chambers. Eight such jars were maintained, a total of 200 individual larvae being observed at each temperature. The green and fresh trifoliate leaves of soybean were offered daily as a larval food. Third instar onwards the individual larvae were transferred to the petri dishes (10 cm diameter), and the food was changed daily till pupation. The instar determination was based on the presence of molted cuticles and overall body size. The development time and mortality of larvae during each instar and the numbers of pupated individuals from the petri plates were recorded daily.

The newly formed pupae (<12 h old) from *S*. *litura* stock colony were isolated and kept individually in test tubes (20 x 3.2 cm size). The test tubes were plugged with absorbent cotton, held in test tube stands and kept inside the growth chambers for incubation at respective temperatures. The number of adults emerging was recorded daily. A total of 100 individual pupae were assessed at each test temperature.

The newly emerged adults (<12 h) from *S*. *litura* stock colony were paired immediately and confined to mating jars (plastic containers of 30 x 15 cm size) covered with black muslin cloth. Thirty jars each with one pair were maintained, thus 30 individual females and males being assessed at each test temperature. The food was provided to the adults in the form of 10% honey soaked cotton wick tied with string hanged from sidewall of the jars. About a week old soybean seedlings raised in small plastic cups were kept inside the moth mating jars as an oviposition substrate for the females. The moths were transferred to the new jars holding fresh potted plants each morning throughout the entire oviposition period of females. The jars holding the plants with egg masses were kept date wise for each female till hatching. The daily numbers of hatched and unhatched eggs were counted from each jar to calculate daily total fecundity per female. The survival time (longevity) was recorded for individual males and females till the death of last adult insect. The sex of progeny was determined at each test temperature by rearing them up to adult emergence to see if the sex ratio varies with temperature conditions.

### Model parameterization

The variability in distribution of development times of immature stages and senescence times of adult stages of *S*. *litura* at various constant temperatures was estimated by fitting a cumulative logit distribution curve [22, 36, 41–44].

$$F\left(x\right)=\frac{1}{\left(1+\text{exp}\left(-\left(ai=b\text{ln}x\right)\right)\right)}$$ (1)

Where, F(x) is the probability to complete development at time x, lnx is the natural logarithm of the days observed, a is the intercept corresponding to temperature i, and b is the common slope of the regression model.

The modified four parameter version of the Sharpe and DeMichele model [45, 46] was used for estimating the rates of immature development and adult senescence in *S*. *litura* at various constant temperatures.

r(T)=p.TTo.[ΔHaR(1To−1T)]e1+exp[ΔHhR(1Th−1T)] (2)

where, r (T) is the development rate at temperature T (°K), R is the universal gas constant (1.987 cal degree^-1^ mol^-1^), p represents the development rate at optimum temperature To (°K) assuming no enzyme inactivation, ΔH_a_ is the enthalpy of activation of reaction catalysed by enzyme (cal mol^-1^), ΔH_h_ is the change in enthalpy at high temperature (cal mol^-1^), and T_h_ is the high temperature at which enzyme is half active.

The mortality in immature life stages of *S*. *litura* at various constant temperatures was estimated by applying Wang model [47].

$$m\left(T\right)=1-\frac{1}{e^{\left\{\left[1+\text{e}\left(-\;\frac{\text{T}-\text{Topt}}{\text{B}}\right)\right]\left[1+\text{ e }\left(-\;\frac{\text{Topt}-\text{T}}{\text{B}}\right)\right]\text{ x H}\right\}}}$$ (3)

Where, m(T) is the rate of mortality at temperature T (°C); Topt is optimum temperature (°C) for cohort survival; B and H are the fitted parameters of equation.

The oviposition was modelled considering the three temperature dependent functions: temperature dependent total fecundity, age-related oviposition frequency and age-specific adult survival. An exponential polynomial equation was fitted to find out the effects of various constant temperatures on total number of eggs produced by a female adult during her whole life span [48, 49].

$$f\left(T\right)=1-\;e^{-\left(aT+bT^{2}+cT^{3}\right)}$$ (4)

Where, f(T) is the total number of eggs produced by the female adult during her entire life span at temperature T (°C); and a, b and c are the fitted equation parameters.

A Gamma function was fitted to describe age-specific fecundity rate of *S*. *litura* females at different constant temperatures [40, 50].

$$f\left(T\right)=\frac{1}{b^{a}X\left(a\right)}X^{a-1}e^{-\left(\frac{T}{b}\right)}$$ (5)

Where, f(T) is the cumulative oviposition frequency at temperature T; X is the normalised age of female expressed as a ratio of age in days and mean survival time; a and b are the fitted equation parameters.

### Statistical analysis and modelling tools

All analyses for developing temperature-dependent phenology model were conducted using ‘*model builder*’ tool in Insect Life Cycle Modelling (ILCYM) software, version 3.0 (International Potato Centre, Lima, Peru) [34]. The best fit model in each case was selected based on well-known goodness of fit indicators such as Akaike’s Information Criterion (AIC) [51] and coefficient of determination (R^2^) along with application of our expert knowledge on biology of *S*. *litura* to predict its life history under a range of environmental temperatures. We then used Analysis of Variance (ANNOVA) and Least Square Design (LSD) as post-hoc tests at *p* = 0.05 significance level for probability thresholds and hypothesis testing in all the regressions to determine which individual traits were governed by these factors.

### Simulation of life table parameters at constant temperatures

Using ‘*stochastic simulation tool’* in ILCYM which is based on rate summation and cohort updating approach [41], the life table parameters *viz*., gross reproductive rate (GRR), net reproductive rate (Ro), intrinsic rate of natural increase (rm), finite rate of increase (ƛ), mean generation time (*T*) and doubling time (*Dt*) were estimated. The process utilised the thermal reaction norms developed for *S*. *litura* life stages at six constant temperatures (15–38°C) for estimating the life table parameters. For determining the sex of emerging adult in simulated life tables, the proportion of male and female individuals at different constant temperatures was considered. For accounting the proportion of females in progeny of simulated life tables, we use two separate oviposition files *i*.*e*. one for female progeny and another for male progeny based on the number of females and males emerged from the populations raised at various test temperatures [34]. We set the initial number of eggs to 100 for the simulations to start at each of the test temperatures (15–38°C) with ten repetitions each. The estimated life table parameters were plotted against respective temperatures and fitted to a polynomial equation [*L*(*T*) = *a*+*bT*+*cT* ^2^, *where L(T) is the respective life table parameter at temperature (T)*].

### Comparison of life tables at constant and fluctuating temperatures

The life table data on *S*. *litura* obtained from fluctuating temperature experiment were compared with model outputs produced using constant temperature experiments. Daily data on minimum and maximum temperatures for each Julian day (average of two years *i*.*e*. 2012 and 2013) obtained from Automatic Weather Station at ICAR-NIASM, Baramati were used as an input for generating the simulations as the simulation procedures in ILCYM is based on daily minimum and maximum temperature data [34]. The annual maximum temperature fluctuated between 23.5 and 41.0°C whereas; the minimum temperature dropped from about 28.2°C in summer (May–June) to ≤ 8.5°C during winter months (December–January). A 15 minute time step length was chosen for accounting diurnal (within-day) temperature variability and temperature-dependent pest population parameters were calculated for each 15 minute time step using a cosine function for half day temperature predictions. For the first half-day temperature predictions, the following equation was used [22, 35].

$$Ti=\frac{\left(Max-Min\right)}{2}\;\times \text{ cos }\left(\frac{\;\times \;\left(\text{i}-0.5\right)}{48}\right)+\frac{\left(Min+Max\right)}{2}$$ (6)

Where, Ti is the temperature (°C) of time step i (i = 1, 2, 3,…48; and Min & Max are the daily minimum and maximum temperatures

Similar procedure was then repeated to estimate *Ti* for the second half day using minimum temperature of the next day in the equation. The stochastic simulation based on rate summation and cohort updating approach [41] was used in estimating life history parameters as described in earlier section. The values for immature development, survival and life history parameters were calculated for each of the above iterations and compared with observed values obtained using fluctuating temperature experiments. The stable age-stage distribution of cohort individuals in a population was plotted. The differences between the predicted and observed life table values of *S*. *litura* were used as a measure for the validity of the model, the significance of which was tested based on *p* value. Differences were treated significant if the value of *p* deviated from zero.

### Spatial simulation and visualization of potential risk

The “*population distribution and risk mapping”* module of ILCYM which is linked to udig, a basic geographic information system (GIS), was used for visualizing the *S*. *litura* potential risk. Using values of the estimated life table parameters the three indices *viz*., establishment risk index (ERI), generation index (GI) and activity index (AI), indicating *S*. *litura* risk at each location were calculated. The following equations were used for estimating the risk indices [22, 34, 35].

ERI = (1−xEgg)×(1−xLarva)×(1−xPupa) (7)

Where, x is a ratio of sum of the number of days in which a single life stage would not survive and a total number of days in a year

GI=  ∑x=1365365Tx365 (8)

Where, Tx is the predicted generation length in days at Julian day x (x = 1, 2,…,365)

$$\text{AI}={\text{log}}_{10}\overset{365}{\underset{x=1}{\prod }}\lambda x$$ (9)

Where, λx is the finite rate of increase at Julian day x (x = 1, 2,…,365)

The simulations were carried out using interpolated baseline climate data obtained from worldclim database (http://www.worldclim.org). The baseline data comprised of derived values of monthly minimum and maximum temperatures interpolated using long term time series from 1950 to 2000 from a global network [52]. For predicting *S*. *litura* response to potential climate warming, the downscaled climate data of SRES-A1B emission scenario for the year 2050 [53, 54] were used to project future temperature changes. The spatial simulations of pest populations were facilitated through grid-based within a defined area according to grid-specific monthly minimum and maximum temperatures from worldclim and CCAFS databases interpolated using the Eq (6) as described above [22, 34]. The risk maps generated were masked with the map (*shape file*) of soybean cultivation areas (*Available online at* http://MapSPAM.info) [55] to obtain the maps that display the *S*. *litura* risk in soybean cultivation areas.

## Results

### Development at constant temperatures

The temperatures within the evaluation range (15–38°C) had a large impact on the development times of *S*. *litura* life stages (Table 1). There were decreases in immature development times from low to high temperatures till 35°C after which development becomes non-linear. The observed mean development times for all the immature life stages were fastest at 35°C (Egg: 2.0 ± 0.03; Larva: 11.0 ± 0.08; Pupa: 7.0 ± 0.04), and lowest at 15°C (Egg: 14.0 ± 0.07; Larva: 93.0 ± 0.3; Pupa: 31.0 ± 1.0). The mean senescence times/ longevities of adults decreased linearly from low to high temperatures within the evaluation range (15°C: Female = 29.0, Male = 27.0; 38°C: Female = 6.0, Male = 5.0). The variability in development times for all the life stages was described by a cumulative logit distribution model (Table 2). The values of coefficient of determination (R^2^) ranged from 0.87 with a slope of 9.22 ± 0.58 for the male adult life stage to 0.95 with a slope of 10.5 ± 0.3 for the egg stage. Values of both the R^2^ and slopes generally declined for adult senescence times (both female and male) as compared with the immature development times.

**Table 1:** Mean development times (days ± SE) of immature stages and senescence times (days ± SE) of adult life stages of *S*. *litura* at different constant temperatures in laboratory.

Temperature (°C)	Egg		Larva		Pupa		Female		Male	
	Predicted	Observed	Predicted	Observed	Predicted	Observed	Predicted	Observed	Predicted	Observed
15	12.71 ± 0.12	14.0 ± 0.07	92.16 ± 0.27	93.0 ± 0.30	29.89 ± 0.16	31.0 ± 1.00	28.30 ± 0.21	29.00 ± 0.23	25.29 ± 0.21	27.00 ± 0.23
20	4.56 ± 0.11	5.0 ± 0.05	26.63 ± 0.12	27.0 ± 0.12	15.60 ± 0.13	16.0 ± 0.13	12.60 ± 0.16	13.00 ± 0.25	11.89 ± 0.18	12.00 ± 0.25
25	3.60 ± 0.06	4.0 ± 0.03	17.52 ± 0.10	18.0 ± 0.12	11.10 ± 0.11	12.0 ± 0.12	7.62 ± 0.11	8.00 ± 0.40	8.16 ± 0.15	8.50 ± 0.35
30	1.93 ± 0.10	2.0 ± 0.06	15.38 ± 0.08	16.0 ± 0.06	8.85 ± 0.09	9.0 ± 0.06	6.94 ± 0.09	7.50 ± 0.22	5.42 ± 0.12	6.00 ± 0.37
35	1.75 ± 0.04	2.0 ± 0.03	10.46 ± 0.06	11.0 ± 0.08	6.07 ± 0.06	7.0 ± 0.04	5.78 ± 0.07	6.00 ± 0.09	4.83 ± 0.08	6.00 ± 0.23
38	2.44 ± 0.03	3.0 ± 0.00	13.16 ± 0.07	14.0 ± 0.06	9.12 ± 0.11	10.0 ± 1.00	5.25 ± 0.07	6.00 ± 0.14	4.23 ± 0.07	5.00 ± 0.07

**Table 2:** Distribution of the cumulative development/ senescence time frequencies for different life stages of *S*. *litura* at various constant temperatures in laboratory (Fitted function: logit model for all stages).

Life stage	a15°C^1^	a20°C	a25°C	a30°C	a35°C	a38°C	Slope (b)	AIC	R^2^
Egg	-26.71 ± 0.85	-15.94 ± 0.50	-13.46 ± 0.44	-6.93 ± 0.25	-5.91 ± 0.28	-9.38 ± 0.40	10.51 ± 0.33	684.24	0.95
Larva	-148.20 ± 5.64	-107.52 ± 4.10	-93.81 ± 3.58	-89.49 ± 3.14	-77.69 ± 2.97	-85.21 ± 3.37	32.76 ± 1.25	292.62	0.94
Pupa	-58.53 ± 2.19	-47.33 ± 1.66	-41.45 ± 1.45	-37.56 ± 1.31	-31.06 ± 1.09	-38.06 ± 1.74	17.23 ± 0.60	271.38	0.92
Female	-44.65 ± 3.09	-33.837 ± 2.34	-27.12 ± 1.90	-25.87 ± 1.81	-23.42 ± 1.65	-22.15 ± 1.56	13.36 ± 0.91	204.55	0.92
Male	-29.77 ± 1.89	-22.82 ± 1.45	-19.35 ± 1.24	-15.58 ± 1.01	-14.52 ± 0.99	-13.29 ± 0.89	9.22 ± 0.58	253.90	0.87

^1^‘a’ represents the intercepts at respective temperatures

**Fig 2:**
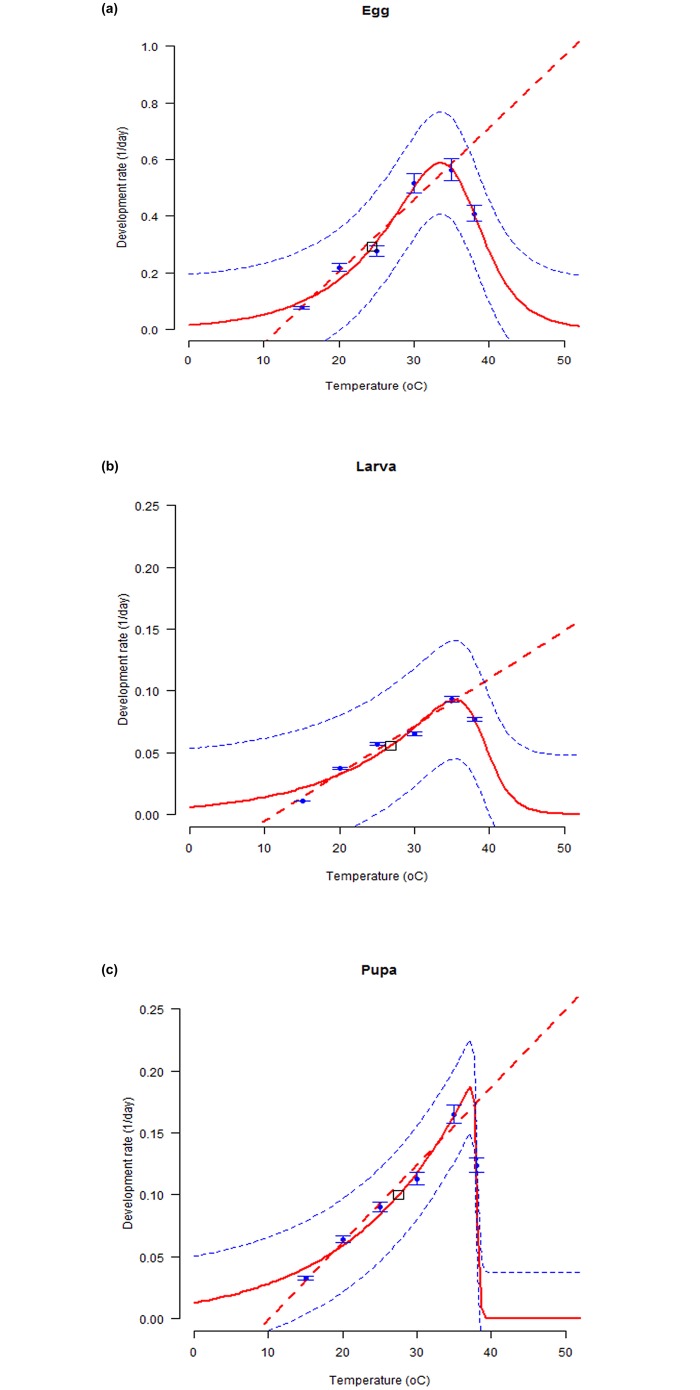
Temperature-dependent developmental rates (1/ day) for immature stages of *S*. *litura*. Egg (a), Larva (b), and Pupa (c). Fitted curves: Modified Sharpe and DeMichele model for all immature stages. The bold solid line is the selected model output and dashed lines above and below represents the upper and lower 95% confidence bands. Bars represent standard deviation of the mean.

**Fig 3:**
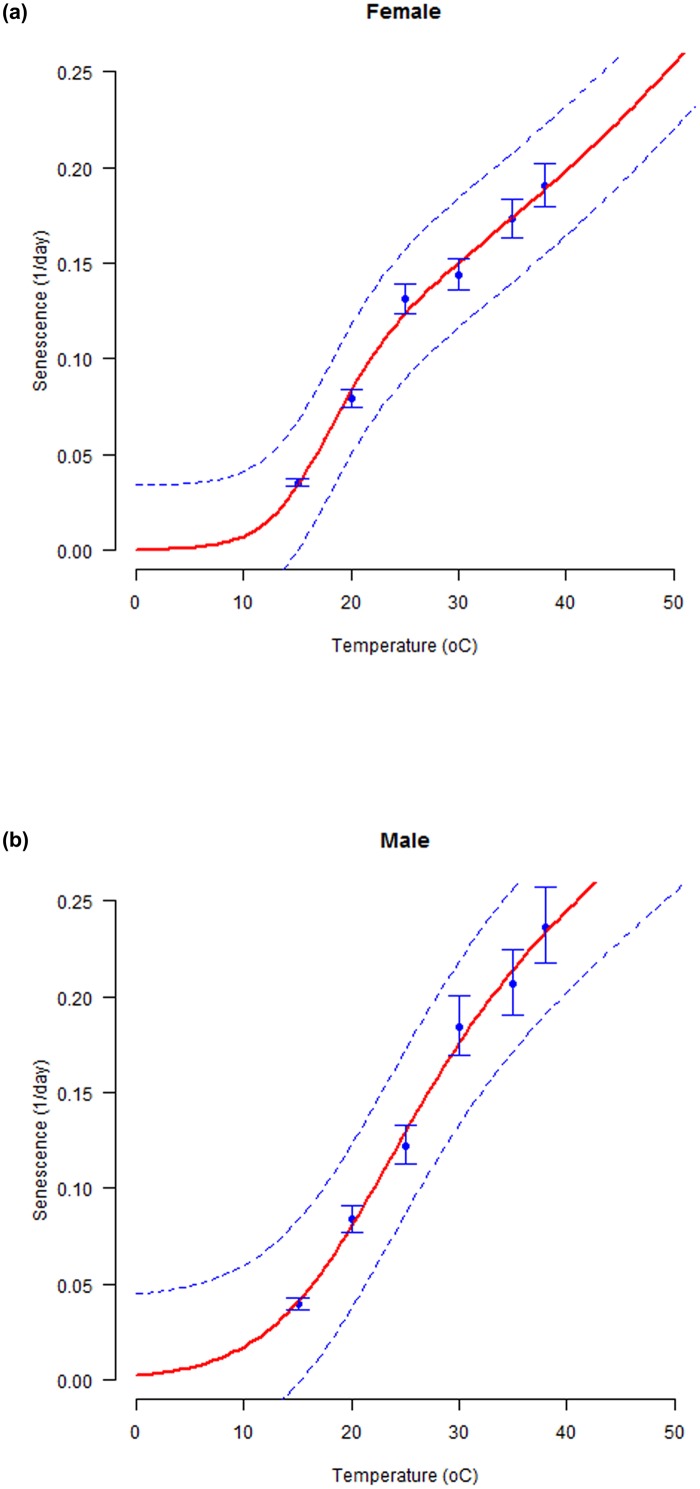
Temperature-dependent senescence rates (1/ day) for adults of *S*. *litura*. Female(a) and Male (b). Fitted curves: Modified Sharpe and DeMichele model for both sexes. The upper and lower 95% confidence intervals of the model are indicated. Bars represent standard deviation of the mean.

Development rate as a function of temperature, increased monotonically for all the immature stages until approximately 34–36°C when it plateaued and then abruptly declined. The thermal reaction norms were well fitted by the modified four parameter version of Sharpe and DeMichele model as indicated by the smallest value of AIC (< -17.0) and highest value of coefficient of determination (> 0.9) for all immature life stages of *S*. *litura* (ANOVA: for egg and pupa, *p* = 0.02; for larva, p = 0.08; df: (3, 2); F statistics: Egg = 33.8; Larva = 10.6; Pupa = 45.5). The lower and upper developmental threshold temperatures predicted for immature life stages of *S*. *litura* were: 10.2°C and 36.3°C (egg), 9.9°C and 38.7°C (larva), and 9.8°C and 38.2°C (pupa). The optimum temperatures estimated by the model for immature development were 24.6°C (297.65°K), 26.7°C (299.67°K) and 26.5°C (299.49) for egg, larva and pupa, respectively (Fig 2 and Table 3). The senescence rate for both the female and the male adults increased linearly from low to high temperatures (Fig 3). The modified four parameter version of Sharpe and DeMichele model provided a good fit to the observed mean senescence rates for both the adult sexes (ANOVA: for both stages, *p* = 0.01; df: (3, 2); F statistics: Female = 91.7; Male = 97.9; R^2^: Female and Male = 0.99). The mean effective temperatures predicted by the model for female and male adults were 17.22°C (290.22°K) and 21.58°C (294.58°K), respectively. The thermal reaction norms thus fitted can be used to predict developmental responses for each life stage of *S*. *littura* over diurnal temperature fluctuations.

**Table 3:** Estimated parameters of the four parameter Sharpe and DeMichele model fitted to the temperature-dependent development rate of immature life stages of *S*. *litura*.

Life stage	P	To	Ha	Hh	Th	AIC	R^2^
Egg	0.29	297.65 ± 1.38	19159.01 ± 0.003	78167.42 ± 0.00	309.34 ± 0.44	-17.84	0.98
Larva	0.05	299.67 ± 5.19	13147.58 ± 3385.80	122073.27± 78.53	311.67 ± 0.99	-33.54	0.94
Pupa	0.10	299.49 ± 1.84	11600.88 ± 0.002	1043640.95 ± 0.00	311.19 ± 0.03	-36.68	0.99

### Immature survival at constant temperatures

**Fig 4:**
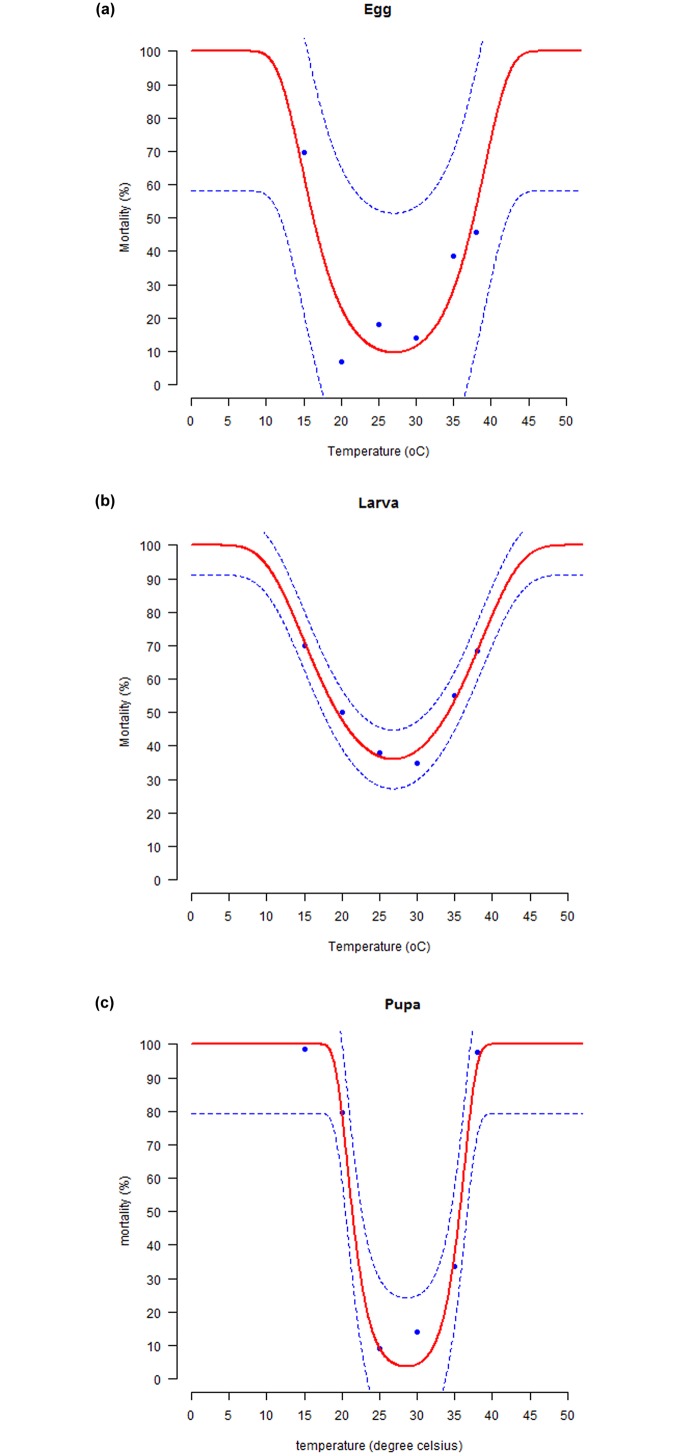
Temperature-dependent mortality rates of immature life stages S. litura. Egg (a), Larva (b) and Pupa (c). Fitted curves: Wang model for all immature stages. The upper and lower 95% confidence intervals of the model are indicated. Markers are observed means, bars represent standard deviation.

Temperature significantly influenced immature survival in *S*. *litura*. Less than 1.5% survival was observed for larval and pupal stages at 38°C. Only two larvae survived until pupation at 38°C, however, they died shortly after pupation. At constant temperature of 15°C, the egg and larval survival ranged between 18–23%, whereas only 1.0% of the pupae could survive to become adults at this low temperature. However, the adults emerged were very small, feeble and inactive, and were died within less than 6 h of emergence. The highest survival of eggs (93.0%), larvae (65.0%) and pupae (91.0%) were observed at 20°C, 30°C and 25°C, respectively. The temperature-dependent mortality rates for all the three immature life stages of *S*. *litura* were well fitted by the Wang model as indicated by the smallest value of AIC (< -6.0) and highest value of coefficient of determination (> 0.91) for all the immature life stages of *S*. *litura* (ANOVA: for all three immature stages, *p* < 0.02; df: (2, 3); F statistics: Egg = 24.08; Larva = 15.02; Pupa = 93.7) (Fig 4 and Table 4).

**Table 4:** Estimated parameters of the Wang model fitted to the temperature-dependent mortality rate for immature life stages of *S*. *litura*.

Life stage	T_o_	B	H	AIC	R^2^	p
Egg	26.18 ± 0.44	2.38 ± 0.45	0.01 ± 0.01	-6.36	0.94	0.01
Larva	26.33 ± 0.73	3.92 ± 0.56	0.10 ± 0.02	-7.00	0.91	0.02
Pupa	28.53 ± 0.24	1.63 ± 0.26	0.01 ± 0.006	-11.44	0.98	0.002

### Adult reproductive traits at constant temperatures

**Fig 5:**
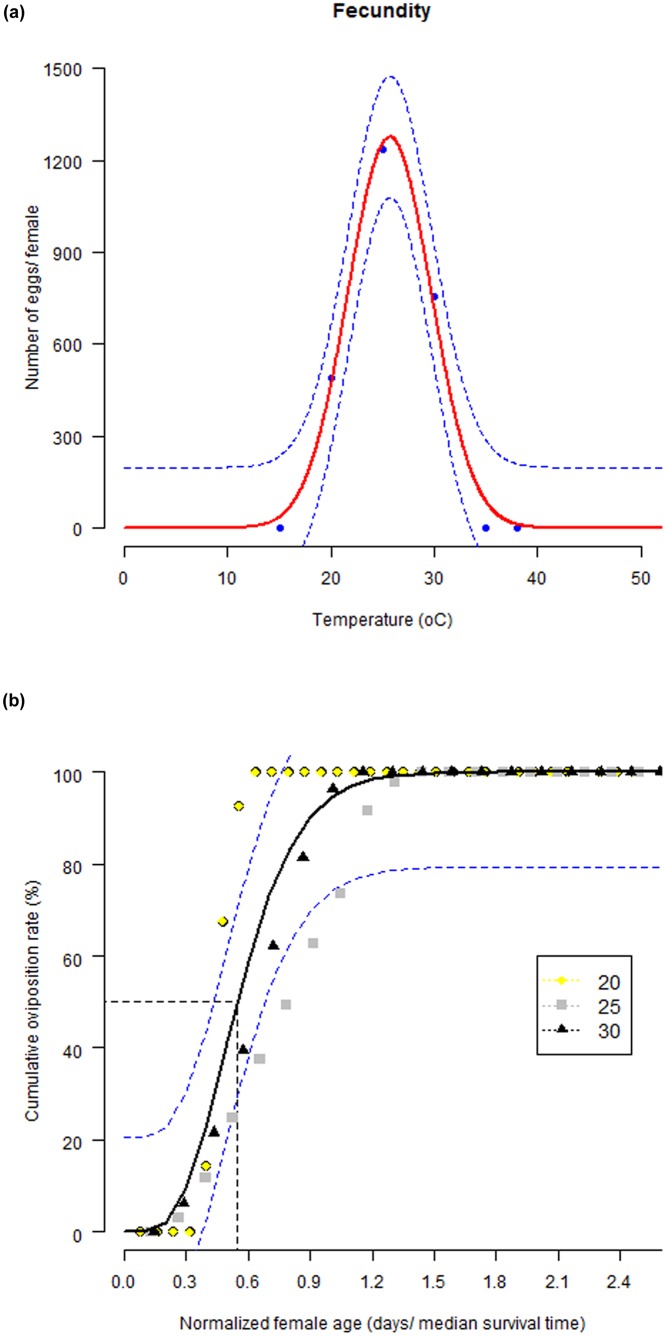
Temperature-dependent reproduction of *S*. *litura*. Total egg production curve, fitted function: exponential polynomial model (a); and Age-related oviposition rate, fitted curve: Gamma distribution function (b). The upper and lower 95% confidence intervals of the model are indicated. The dots are observed data points.

The temperature influenced significantly the reproductive traits in *S*. *litura*. Eggs were produced by females from temperature regimes between 20–30°C only, with the minimum number of total eggs per female at 20°C (492.3) and peak egg laying at 25°C (1234.9) in their gonotrophic cycle. No egg laying occurred at 15°C and 38°C temperatures, indicating that the range of temperatures tested encompassed both the minimum and maximum thresholds for egg production for *S*. *litura* population. Accordingly, a significant effect of temperature on mean total fecundity was identified by fitting exponential polynomial function (Fitted equation: *f(T)* = e(-13.30+1.59T-0.03T^2^); ANOVA: *p* = 0.0008; df = (2, 3); F statistic = 167.3; R^2^ = 0.99) (Fig 5a). The relationship between cumulative oviposition rate and female age was well described by the Gamma function (Fitted equation:$f\left(T\right)=\frac{1}{10.32^{5.99}X\left(5.99\right)}X^{5.99-1}e^{-\left(\frac{T}{10.32}\right)}$; P < 0.001; df = (1, 91); F = 817.6; R^2^ = 0.90) (Fig 5b). Fifty per cent of the total eggs were laid by the female at the physiological age of 0.55.

### Life table parameters at constant temperatures

**Fig 6:**
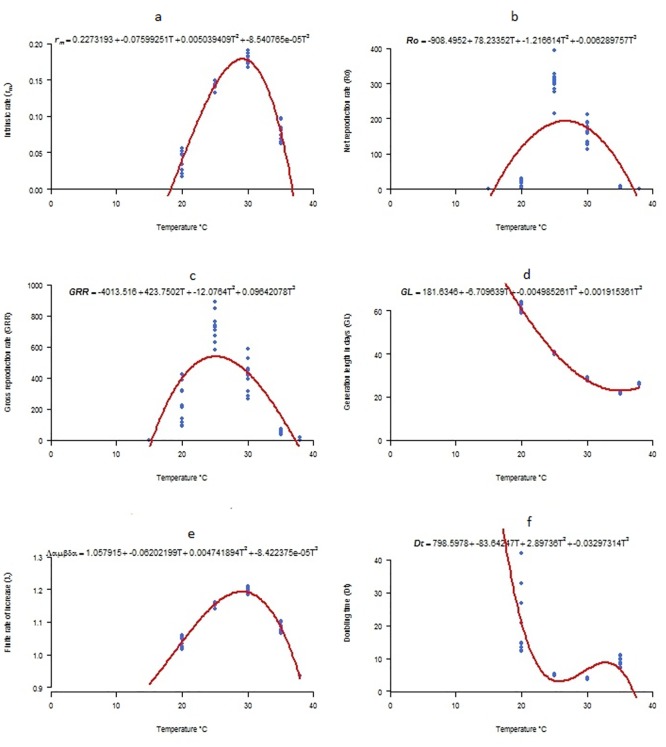
Life table parameters of *S*. *litura* estimated at six constant temperatures. Intrinsic rate of natural increase (a), Net reproduction rate (b), Gross reproductive rate (c), Mean generation time (d), Finite rate of increase (e), and Doubling time (f).

*S*. *litura* population attained a maximum net reproductive rate (349.4±16.8 females/female/generation) at 25°C temperature. The total fecundity was also maximal (824.5±40.7 individuals/female/generation) at this temperature. The suitable range for *S*. *litura* reproduction was observed between 20–30°C. Intrinsic rate of increase (rm) and finite rate of increase (λ) were maximum at 30°C with values of 0.18 ± 0.002 and 1.2 ± 0.003, respectively. Values estimated for ‘T’ indicate that the mean length of generations decreased with increase in temperatures from 59.1 ± 0.45 days at 20°C temperature to 21.8 ±0.12 days at 30°C. Shortest doubling time was observed at 30°C (3.8 ± 0.05 days). Fitting of a polynomial model to the estimated life table parameters predicted temperatures between 25–30°C as a favourable range for *S*. *litura* development, survival and reproduction, where high reproductive potential and shorter generation length were observed (Fig 6).

### Comparison of life tables at constant and fluctuating temperatures

**Fig 7:**
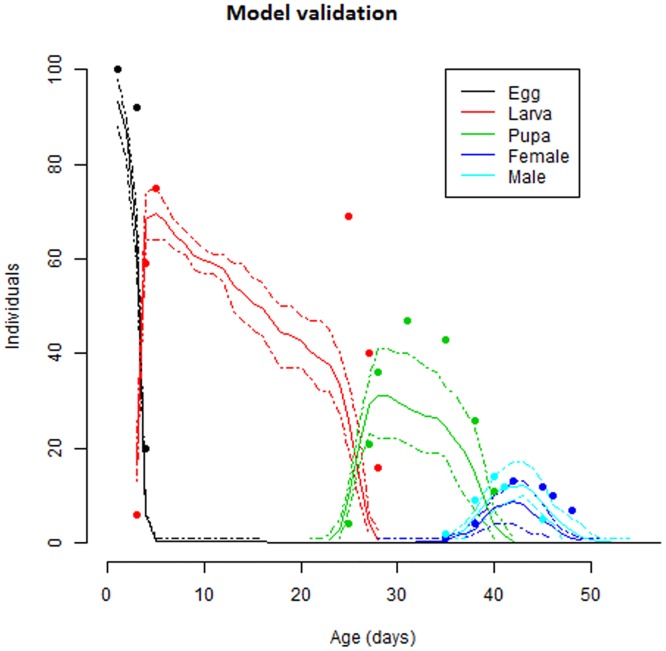
Model validation. Observed and simulated life stage frequencies of *S*. *litura*. Dots represent observed data points at fluctuating experiments, and the lines represent developmental frequencies simulated at fluctuating temperatures based on thermal reaction norms for constant temperatures.

The range of diurnal temperature fluctuations used in generating life table data for *S*. *litura* extended between 21.7–30.2°C with a mean temperature of 25.5 ± 0.4°C. Thus, daily temperature fluctuations ranged between 3.8°C below, and 4.7°C above the mean temperature. However, it did not reach 38°C, where high mortality and adverse developmental effects were observed in the constant temperature treatments. The treatment of daily temperature fluctuations approximated the developmental effects of constant temperature of 25°C. The developmental effects of diurnal temperature variations predicted from the models that were established based on thermal reaction norms designed for constant temperatures were all qualitatively similar to those observed in our fluctuating temperature experiments for immature life stages of *S*. *litura* (Table 5 and Fig 7). Only the lack of concordance was quantitative, with larger than predicted development times for egg and larval stages and smaller than predicted development times for pupal life stage. The developmental times and reproductive attributes observed under fluctuating temperatures and those predicted by simulating fluctuating temperature effects using constant temperature reaction norms were nearly approaching with the life table parameters observed at 25°C in constant temperature experiments. For both the predicted and observed cases, the largest effects of diurnal temperature fluctuations occurred when immatures experienced extreme temperatures as revealed by increased immature mortality. In contrast to the immature developmental times and mortality, the predicted effects of temperature fluctuations on *S*. *litura* life history parameters were quite different from those actually observed. The largest discrepancy is generated in life history parameters relevant to reproduction due to an unexpected decrease in net reproductive rate (Ro) and increase in gross reproductive rate (GRR) than predicted. The discrepancies between observed and predicted effects of diurnal temperature fluctuations on rest of the life history parameters *i*.*e*. intrinsic rate of natural increase (rm), finite rate of natural increase (ƛ) mean generation time (T), and population doubling time (Dt) were similar to those seen in development response and quite different from Ro and GRR.

**Table 5:** Comparisons between the developmental effects of diurnal temperature fluctuations predicted from models based on thermal reaction norms designed for constant temperatures with those observed in fluctuating temperatures.

Parameter	Predicted values	Observed values	*p*
**Development time** (days)			
Egg	2.87 ± 0.14	3.13	0.0001
Larva	22.22 ± 0.34	23.94	0.0001
Pupa	12.90 ± 0.39	11.43	0.0001
Mortality (%)			
Egg	28.00 ± 0.10	25.00	0.08
Larva	51.70 ± 0.10	37.30	0.0001
Pupa	45.10 ± 0.15	40.40	0.10
**Life history parameters**			
Net reproductive rate (*R*o) (♀/♀)	76.72 ± 46.51	91.84	0.14
Gross reproduction rate (*GRR*) (♀/♀)	381.68 ± 181.01	331.37	0.33
Intrinsic rate of increase (*r*_m_)	0.10 ± 0.02	0.11	0.11
Finite rate of increase (λ)	1.10 ± 0.02	1.11	0.11
Mean generation time (*T*) (days)	42.03 ± 0.76	42.11	0.62
Doubling time (*Dt*) (days)	6.88 ± 1.14	6.46	0.10

### Spatial mapping: changes in *S*. *litura* distribution and abundance in response to temperature change

**Fig 8:**
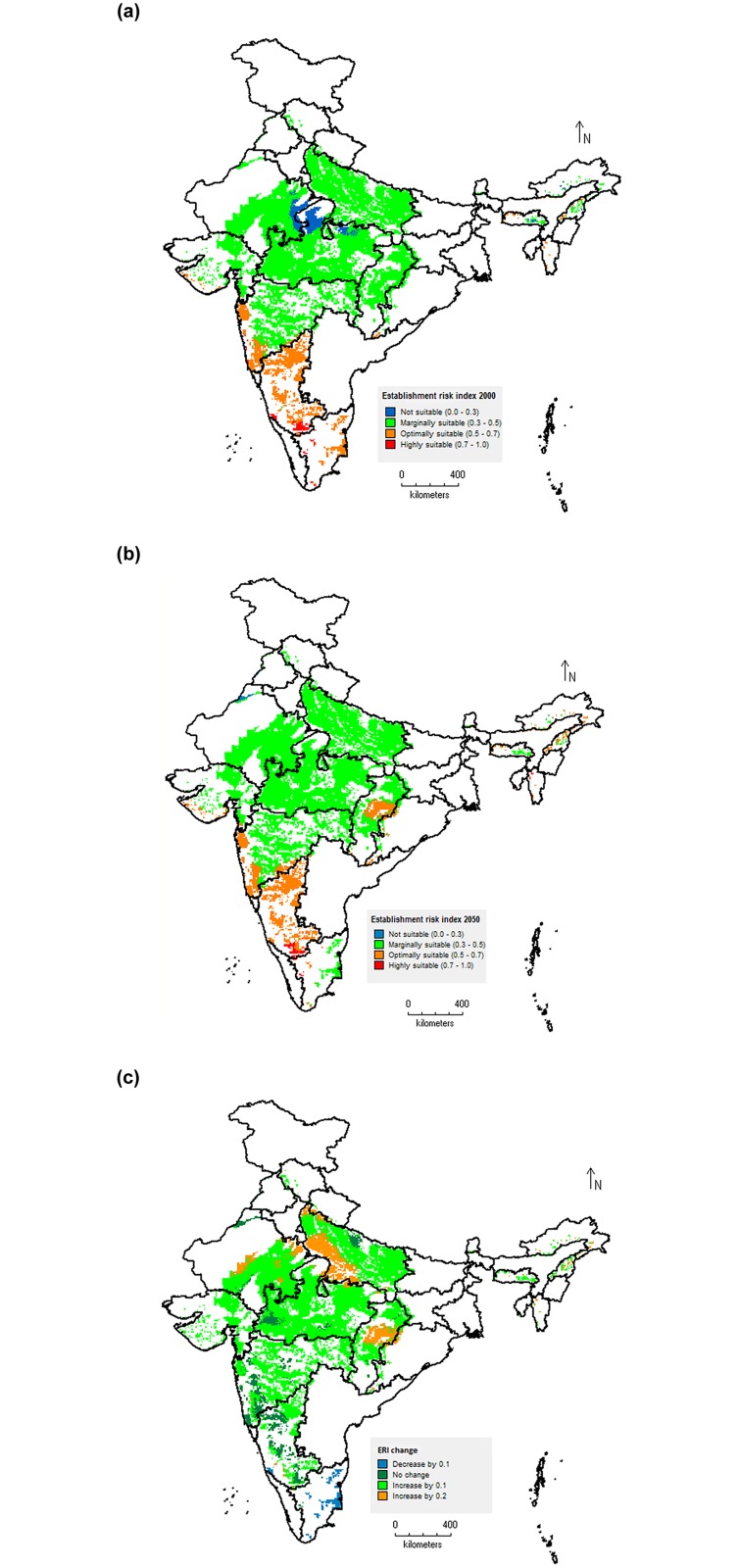
Change in establishment and future distribution of *S*. *litura* in soybean growing areas of India based on establishment risk index (ERI). Current climatic conditions (a), Future climatic conditions (b), and Absolute change in ERI (c). Geographical regions having ERI values > 0.6 are associated with the risk of permanent establishment.

Mapping the values of ERI, GI and AI for current (baseline worldclim climate data for the year 2000) and future temperature conditions (SRES A_1_B scenario for the year 2050), and the absolute change in these indices between current and future temperatures reveal substantial differences in pest status of *S*. *litura* over time. From the maps prepared using baseline temperature data (year 2000), it is clear that the ERI reflected well the distribution of *S*. *litura* under present climatic conditions in major soybean cultivation area of India (Fig 8a). The map shows that areas in states like entire Madhya Pradesh, Maharashtra, Rajasthan, Chhattisgarh and Uttar Pradesh which altogether constituted about 90% of soybean cultivation area of India, are predicted as marginally suitable areas for establishment of *S*. *litura* (ERI value 0.3–0.5). The entire areas in states like Tamil Nadu and Karnataka, and coastal regions of Maharashtra and Gujarat states were predicted optimally suitable habitats for establishment and survival of *S*. *litura* (ERI value 0.5–0.7). Under future climate scenario of 2050, majority of the soybean areas are predicted to become more suitable for *S*. *litura* establishment and survival over time due to northward movement in the climatically suitable area for the pest (Fig 8b). This will be leading to increase in ERI value by 0.1, and is reflected well by absolute change in ERI (Fig 8c). The western part of Uttar Pradesh state, Raipur, Janjgir Champa and Durg districts in Chhattisgarh state, and Jodhpur, Nagaur and Jhunjhnu districts of Rajasthan state are predicted with an increase in suitability index for establishment by 0.2. Rest of the soybean area where no change is predicted under future climate will remain at least moderately climatically suitable for the *S*. *litura* and this coincides reasonably well with suitable areas for GI and AI.

**Fig 9:**
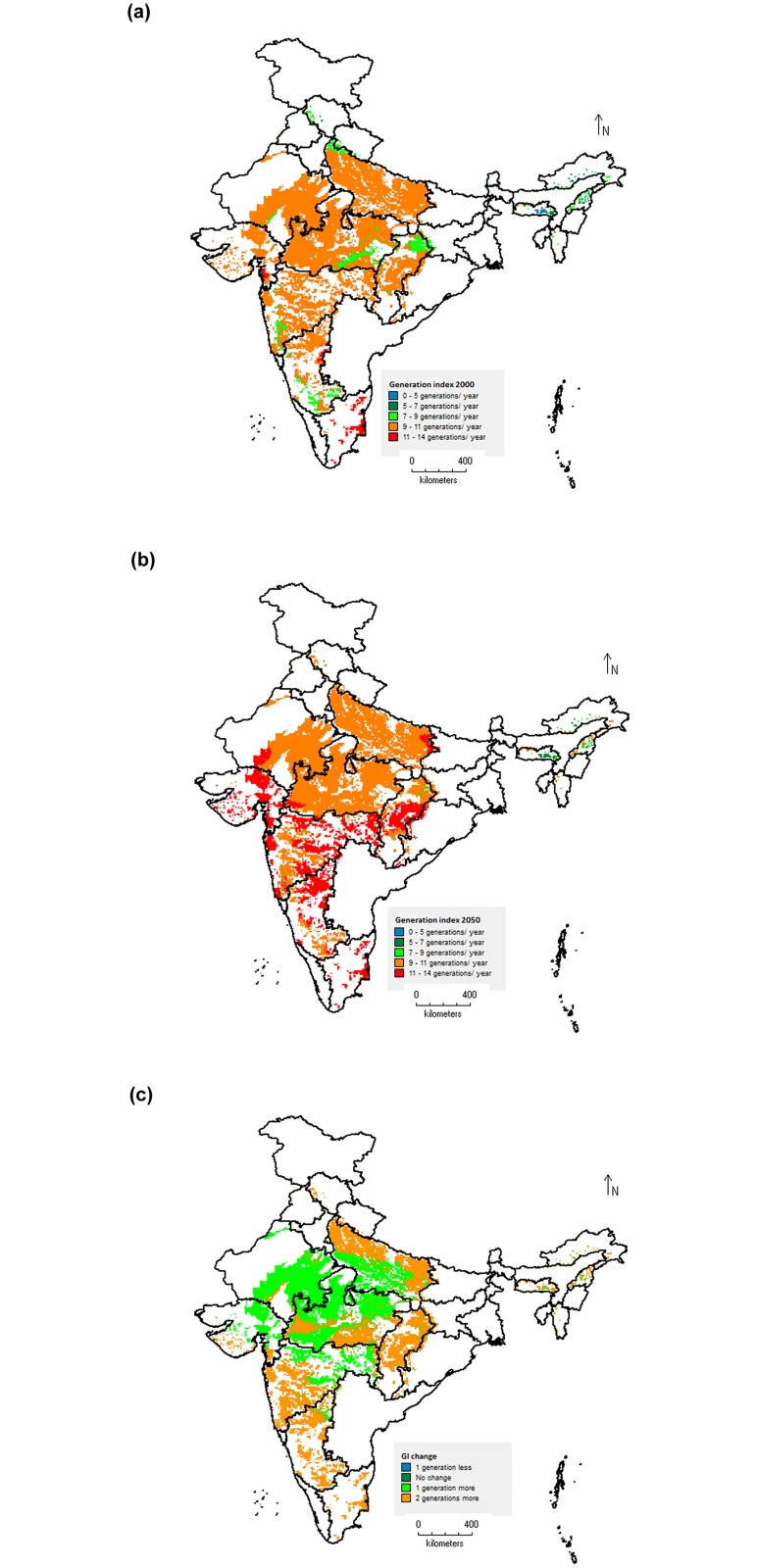
Change in number of generations per year of *S*. *litura* in soybean growing areas of India based on generation index (GI). Current climatic conditions (a), Future climatic conditions (b), and Absolute change in GI (c). Economic damage is most likely to occur in the regions with generation index values > 7.0.

**Fig 10:**
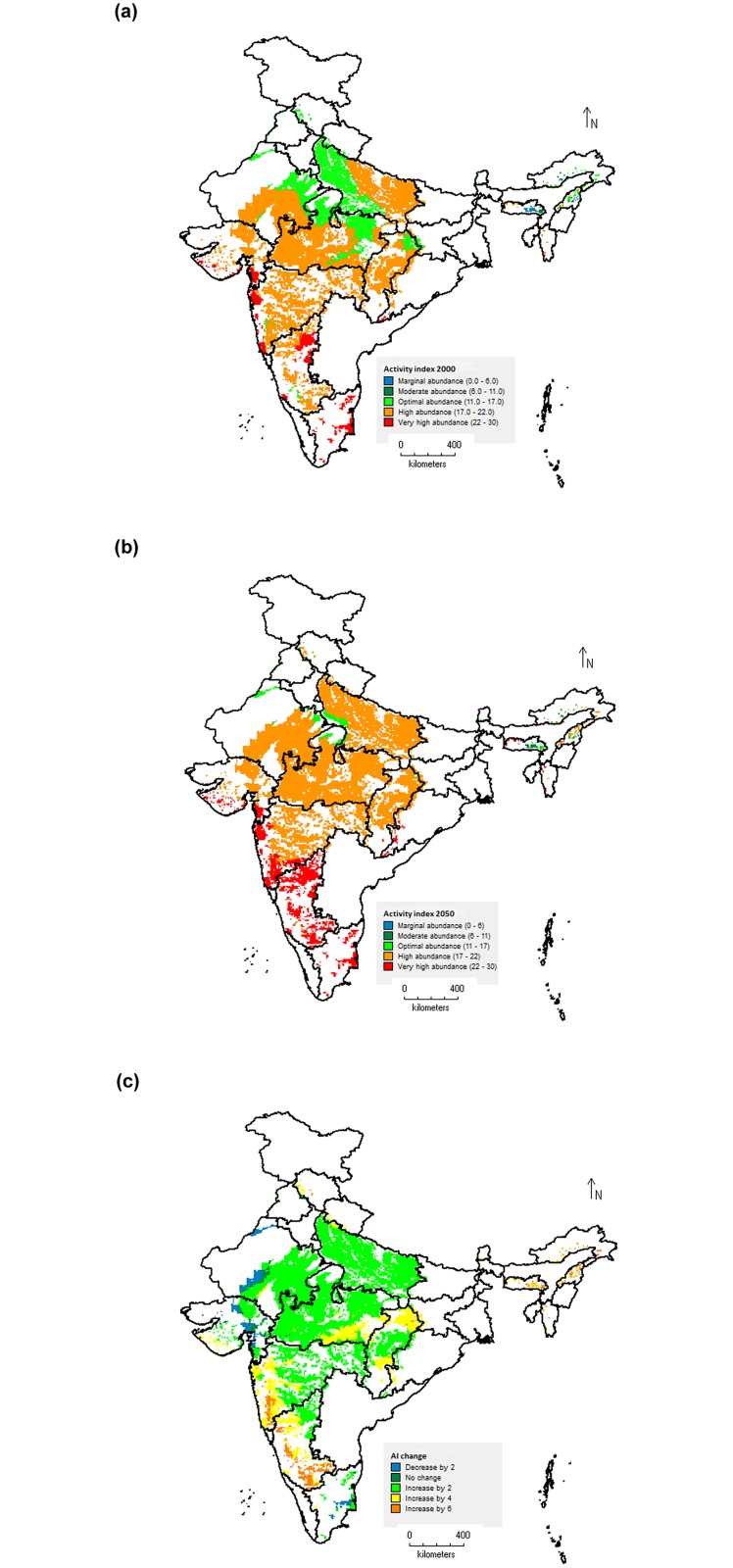
Change in abundance and damage potential of *S*. *litura* in soybean growing areas of India based on activity index (AI). Current climatic conditions (a), Future climatic conditions (b), and Absolute change in AI (c). An index value of 1 represents 10-fold potential population increase within a year. *e*.*g*.: An AI value of 4.0 represents 10^4^ *i*.*e*. 10,000 times potential population increase within a year.

The maps of GI indicate the mean numbers of generations that can be produced per year by *S*. *litura* under the given temperature conditions in soybean cultivation areas of India. The map prepared using baseline temperature data revealed that under present temprature conditions, *S*. *litura* is capable of producing about 9–11 generations in a year in majority of the soybean cultivation areas of India. The highest numbers of 11–14 generations per year are predicted for Tamil Nadu state (Fig 9a). Under future temperature scenario of 2050, an average increase of 1.0–2.0 generations per year is expected throughout the soybean cultivation areas of India (Fig 9b and 9c). The entire areas in Tamil Nadu and Chhattisgarh states, majority parts of Karnataka state, Central and Southern parts of Maharashtra state, Coastal part of Gujarat state and North-western part of Uttar Pradesh state are likely to experience an increase of 2.0 more generations per year. The current temperature conditions throughout the soybean cropping area of India were predicted at least optimally suitable habitats for *S*. *litura* population abundance (AI value > 11.0). Approximately, 80% of the soybean area is predicted as highly suitable under present temperature conditions with AI value > 17.0 (Fig 10a). Under the future temperature conditions (2050 scenario), *S*. *litura* abundance and damage activities will be increased significantly (Fig 10b). The map of absolute AI change indicated that majority of the areas will experience an increase of activity index by a value of 2.0 (Fig 10c). The maximum increase of population abundance by a factor of 6.0 is predicted in the parts of Karnataka and Maharashtra states. The parts of Tamil Nadu and Rajasthan states where decreased suitability for establishment and survival is predicted under future temperature conditions of 2050 (map of ERI change, Fig 8c) are coinciding reasonably well with decreased suitability for pest abundance (map of AI change, Fig 10c).

## Discussion

Here, we used process-based phenology modelling approach to describe the temperature-dependent population growth potential of the common cutworm *S*. *litura* reared on soybean leaves in laboratory. Our study revealed that *S*. *litura* could sustain under constant temperatures ranging between 20°C and 30°C, however favourable temperature range observed was only between 25°C and 30°C. We also found that the females of *S*. *litura* were unable to lay eggs at constant temperatures of 15°C, 35°C and 38°C; as a result we could not estimate population growth parameters at these temperatures. Our results are in larger agreement with those reported by Rao et al [28], who also did not get *S*. *litura* oviposition at constant high temperatures of 35°C and 37°C, however, only deviation that existed for low temperature of 15°C, where they reported egg laying. The studies by Miyashita [24] and Rao et al [28] on the developmental effects of constant and alternating temperatures on *S*. *litura* addressed only the development rates and estimation of thermal constants. However, they did not consider the temperature-dependent immature mortality, adult senescence and female fecundity which are considered highly important in understanding pest population dynamics [33, 40]. Rest of the studies that deal with estimating *S*. *litura* life table parameters were conducted using only single constant temperature [25–27, 29–31]. In the present study, we evaluated the effects of ecologically relevant range of daily temperature fluctuations compared with effects of constant temperatures in a regime of 15–38°C on development, survival and reproduction of *S*. *litura*. Thus, our predictions have taken into account the whole life history for estimating *S*. *litura* population growth potential at various temperatures.

The lower developmental threshold temperatures (LTTs) for *S*. *litura* immature stages estimated in the present study (egg: 10.2°C, larva: 9.9°C, pupa: 9.8°C) were in closer agreement with the earlier reports [24, 28]. The slight deviations that exist among the reported LTTs may be attributed to differences in rearing conditions and host plants used as larval food [56]. Unlike previous studies, in addition to LTTs, we estimated the optimum temperatures for development (*To*) and the higher developmental threshold temperatures (HTTs) for the immature life stages through fitting of non-linear functions of higher biological significance.

The developmental durations of *S*. *litura* immature stages observed at different constant temperatures were largely in conformity with earlier reports [24, 25, 28, 31]. The developmental durations of larva (17.1 days), pupa (8.4) and adults (6.1) of *S*. *litura* reared on soybean at single constant temperature of 27 ± 0.5°C [25] were in proximity to our results obtained at constant temperatures of 25°C and 30°C. However, slightly longer developmental durations were reported for *S*. *litura* larvae (23–25 days) when reared on tobacco leaves in laboratory at 26 ± 1.0°C [31, 56]. Such deviations may be attributed to the different host plants used as food for rearing the insect species [56].

The survival rates of *S*. *litura* immature stages varied significantly at various constant temperatures. The constant temperatures below 20°C and above 35°C were highly unfavourable for survival of all the immature life stages, where increased mortality rates were observed. In the present study, we could obtain highest percentage of survival (65.0%) in *S*. *litura* larva when reared at constant temperature of 30°C in laboratory. Manimanjari et al. [57] reported the similar trend in survivorship of *S*. *litura* wherein they found lowest and highest survival at 20°C and 30°C temperatures, respectively when reared on sunflower (*Helianthus annus* L.). Further, the survival rates of immature stages of *S*. *litura* are highly dependent on host plants used for larval feeding [56].

At higher constant temperature (> 35°C), the life span of female adults of *S*. *litura* was approximately 4–5 times less than life span at low constant temperature (15°C), leading to substantial shortening of reproductive phase. The lifetime fecundity and cumulative oviposition rate of *S*. *litura* were found highly temperature-dependent. A curvilinear response was observed for fecundity with a maximum at 25°C (1,234.9 eggs/ female) and decreasing at temperatures below and above this temperature. This demonstrates that prevalence of optimum temperature can play a bigger role in determining the suitability of climate for the mating and oviposition of *S*. *litura* adults. Fairly similar trend in temperature-dependent fecundity of *S*. *litura* was reported by earlier workers, who found temperatures between 25–30°C as suitable range for *S*. *itura* reproduction, and temperatures > 33°C were highly detrimental [25, 26, 57, 58]. The total number of eggs laid per female in our study was slightly lower than those reported in literature [25, 26, 57, 58]. This can be ascribed to the differential ovipositional response of *S*. *litura* when reared on different host plants [56]. Oviposition and its rate are crucial components on which depends an insect population dynamics and hence detailed knowledge on temperature-dependent age-specific fecundity is imperative for developing pest forecasting models [33]. This study presents only the effect of temperature on *S*. *litura* fecundity when reared on soybean leaves. However, fecundity is also influenced by several other factors such as host nutritional quality, food availability, nutrition of immature stages, and abiotic factors like light intensity, relative humidity, etc. [3, 5, 30, 31, 58, 59]. Thus, present results represent only the potential fecundity of *S*. *litura*.

The effects of constant temperatures on the life table parameters of *S*. *litura* have been reported in many studies [5, 29, 30, 31, 57], however only one of them [57] addressed a range of constant temperatures within the ecologically relevant limits for *S*. *litura* development. Manimanjari et al. [57] reported slightly higher values for the life table parameters of *S*. *litura* reared on sunflower at six constant temperatures between 20–35°C. Our results are largely consistent with literature reports, however, discrepancies occurred between r_m_ and Ro (r_m_ is a function of Ro) predicted from this study and those reported in literature. It seems that these discrepancies are largely due to the deviations from the selected sub-model for development and mortality of immature stages and total fecundity per female, which are considered to be the most variable factors [22, 35, 40]. Besides, host material *i*.*e*. soybean leaves used in rearing *S*. *litura* adds further to the variability in life cycle [36, 56]. Such a difference in phenology model formulation may be responsible for the overall discrepancy between the results presented here and those reported by previous authors.

The comparison of the maps of ERI with the literature data available on the known distribution of *S*. *litura* in soybean growing area of India [2, 4] indicated that present study predicted the marginal to optimal suitability of soybean growing areas for distribution and survival of *S*. *litura* under current climatic conditions. The generation indices simulated were reasonably predicted when compared with the literature data. *S*. *litura* is reported to complete about 12 generations in a year on groundnut crop in Andhra Pradesh state of India [4]. By inputting the thermal requirements of 551.20 DD, and lower and upper threshold temperatures of 10.5°C and 37.0°C in CLIMEX, Sridhar et al. [60] reported that under current climatic conditions, *S*. *litura* can produce as many as 11–12 generations per year in coastal areas of Andhra Pradesh, Tamil Nadu and Kerala; 9–10 generations in central India, and less than 9 generations per year in far northern and north-eastern states of India. Further, they reported an overall increase of 1–2 generations under future climate scenario for the year 2030. These findings greatly support our predictions on *S*. *litura* number of generations per year under both current and future climatic scenarios. The comparisons of activity indices estimated for *S*. *litura* with literature reports on its incidence [8, 15, 17] reasonably represented its potential population growth and damage activity in various geographic areas of India. In Madhya Pradesh state, the pest has been reported to cause soybean yield losses in a range of 26–29% [8], whereas yield losses worth USD 22.5 crores have been reported from Maharashtra state [15, 17]. These regions are represented on AI map as highly suitable areas for pest activity (AI value >17).

The present predictions are based on the effects of only temperature, a key abiotic factor affecting growth, survival and reproduction of poikilotherms like insects [18]. The effects of other abiotic (humidity, light, rainfall, etc.) and biotic (parasitoids, predators, microorganisms, etc.) factors influencing the pest population abundance are not considered in this study. Hence, the model outputs represent only the potential population growth parameters for *S*. *litura* in a given agro-ecological region. Thus, it needs to be cautiously interpreted while predicting field dynamics and abundance of *S*. *litura* population, where abiotic and biotic factors other than temperature do come to play the role in regulating pest population dynamics. However, the authors are of opinion that if coupled with field observations, the model can certainly contribute to the improved understanding of the field dynamics and the activity of *S*. *litura* under a range of temperatures. In addition to the suitability of climatic factors, the availability of host plant has a substantial impact on survival and population dynamics of insect species that have narrow host range [61]. However, for generalist herbivores, like *S*. *litura*, their ability to have sustained population growth on a wide range of host plant species makes the role of availability of one or few among the many host plants less significant in determining their geographic distribution and abundance [62, 63]. Furthermore, all other possible sources of uncertainty (*e*.*g*. predictions of climate change, future host plant distribution, etc.) in making the predictions on *S*. *litura* distribution, abundance and its response to future climatic changes warrant more detailed insights into the abiotic and biotic factors that impact the species population growth and spatio-temporal abundance. This will form a basis for formulating next part of our study on abiotic-biotic interaction model for *S*. *litura* using the information on temperature-dependent phenology.
